# Supplementary material for: The interactome of intact mitochondria by cross-linking mass spectrometry provides evidence for coexisting respiratory supercomplexes
Source: Mol Cell Proteomics. 2017 Dec 8;17(2):216–32. doi: 10.1074/mcp.RA117.000470 (PMC5795388; doi:10.1074/mcp.RA117.000470)
Supplement: Supplemental Data [file supp_RA117.000470_133922_0_supp_23978_fzffwf.zip › spectra_annotation/mito_spectra_annotation/23-1-2-1-5-1.pdf]

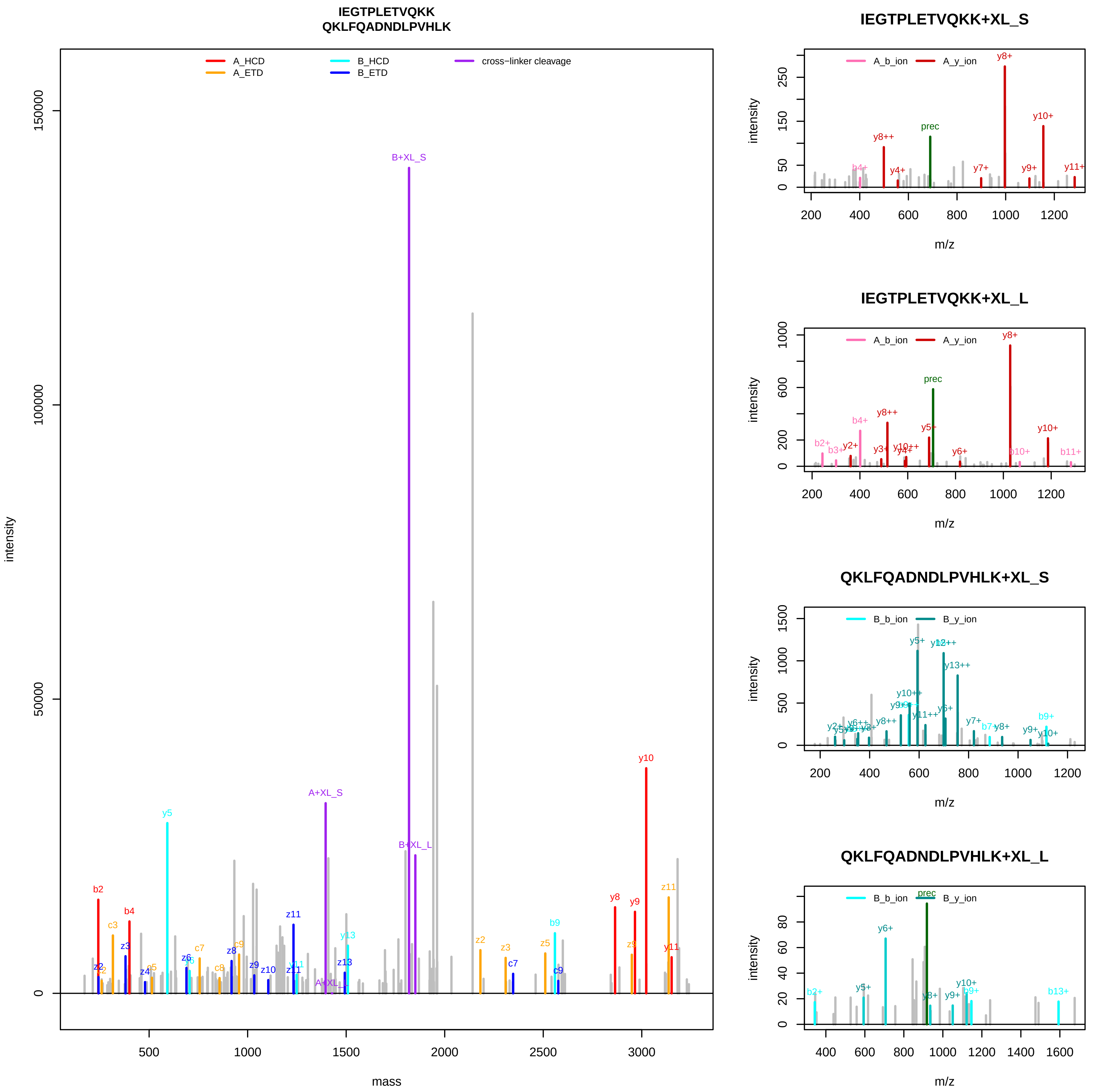

IEGTPLETVQKK
QKLFQADNDLPVHLK
A_HCD
A_ETD
B_HCD
B_ETD
cross−linker cleavage
intensity
mass
B+XL_S
A+XL_S
B+XL_L
A+XL_L
b2
b4
c3
z2
z3
z4
c5
y5
z6
c7
c8
z8
c9
z9
z10
z11
y11
z13
y13
z2
z3
c7
z5
b9
c9
y8
y9
z9
y10
z11
y11
IEGTPLETVQKK+XL_S
A_b_ion
A_y_ion
prec
m/z
IEGTPLETVQKK+XL_L
QKLFQADNDLPVHLK+XL_S
B_b_ion
B_y_ion
QKLFQADNDLPVHLK+XL_L
